# CCAAT Enhancer Binding Protein and Nuclear Factor of Activated T Cells Regulate HIV-1 LTR via a Novel Conserved Downstream Site in Cells of the Monocyte-Macrophage Lineage

**DOI:** 10.1371/journal.pone.0088116

**Published:** 2014-02-14

**Authors:** Satinder Dahiya, Yujie Liu, Michael R. Nonnemacher, Will Dampier, Brian Wigdahl

**Affiliations:** Department of Microbiology and Immunology, and Center for Molecular Virology and Translational Neuroscience, Institute for Molecular Medicine and Infectious Disease, Drexel University College of Medicine, Philadelphia, Pennsylvania, United States of America; University of Nebraska Medical Center, United States of America

## Abstract

Transcriptional control of the human immunodeficiency virus type 1 (HIV-1) promoter, the long terminal repeat (LTR), is achieved by interactions with cis-acting elements present both upstream and downstream of the start site. *In silico* transcription factor binding analysis of the HIV-1 subtype B LTR sequences revealed a potential downstream CCAAT enhancer binding protein (C/EBP) binding site. This binding site (+158 to+172), designated DS3, was found to be conserved in 67% of 3,858 unique subtype B LTR sequences analyzed in terms of nucleotide sequence as well as physical location in the LTR. DS3 was found to be well represented in other subtypes as well. Interestingly, DS3 overlaps with a previously identified region that bind members of the nuclear factor of activated T cells (NFAT) family of proteins. NFATc2 exhibited a higher relative affinity for DS3 as compared with members of the C/EBP family (C/EBP α and β). DS3 was able to compete efficiently with the low-affinity upstream C/EBP binding site I with respect to C/EBP binding, suggesting utilization of both NFAT and C/EBP. Moreover, cyclosporine A treatment, which has been shown to prevent dephosphorylation and nuclear translocation of NFAT isoforms, resulted in enhanced C/EBPα binding. The interactions at DS3 were also validated in an integrated HIV-1 LTR in chronically infected U1 cells. A binding knockout of DS3 demonstrated reduced HIV-1 LTR-directed transcription under both basal and interleukin-6-stimulated conditions only in cells of the monocyte-macrophage lineage cells and not in cells of T-cell origin. Thus, the events at DS3 positively regulate the HIV-1 promoter in cells of the monocyte-macrophage lineage.

## Introduction

Human immunodeficiency virus type 1 (HIV-1) is transcriptionally regulated by cellular and viral proteins interacting with the cis-regulating elements in the viral promoter, the long terminal repeat (LTR). The HIV-1 LTR is approximately 640 base pairs in length and is divided into three components, the unique 5′ (U5), unique 3′ (U3), and the repeat (R) regions [1]. The HIV-1 proviral DNA regulatory elements present upstream of the transcription start site (+1), constituting a typical RNA polymerase II promoter, have been studied extensively with regard to HIV-1 transcriptional regulation, as previously reviewed [2], [3]. With the constant evolution in our understanding of the nucleosome packaging within the HIV-1 LTR [4], [5], the contributions of transcription factor (TF) binding sites (TFBSs) located downstream of the start site have been examined previously [6]–[9]. In this regard, the downstream binding sites for AP-1, AP3-like, DBF1 (dehydration-responsive element [DRE]-binding factor), and Sp1 (specificity protein 1) have been previously demonstrated to regulate basal transcription within the context of the integrated LTR [7]. The relative importance of these sites depends on the cellular phenotype of the infected cell with respect to cell type and activation and differentiation state, as the subset of TFs available at a given point in time is regulated by both the differentiation status of the cell and the activation signals received by a given cell population.

Three CCAAT enhancer binding protein (C/EBP) binding sites have been previously described within the upstream HIV-1 LTR: upstream site 1 (US1), located immediately 5′ of the distal nuclear factor-κB (NF-κB) binding site (−105 to −116); upstream site 2 (US2), located upstream of US1 (−164 to −173); and upstream site 3 (US3), located upstream of US2 (−245 to −253) [10]–[13]. Consensus sites for HIV-1 subtype B have been identified for US1 and US2, while a consensus binding site has not been reported for US3 [10]. The binding sites US1 and US2 have been shown to be necessary for HIV-1 expression and replication in cells of the monocyte-macrophage lineage, but they were shown to be dispensable in T-cell lines and primary T-cell populations [11]. However, the existence of functional downstream C/EBP binding site(s) in the HIV-1 LTR has not been previously identified.

C/EBPs are a bZIP (basic leucine zipper) family of transcription factors that includes C/EBPα, C/EBPβ, C/EBPγ, C/EBPδ, C/EBPε, and C/EBPζ (CHOP10 [C/EBP homologous protein 10]/GADD153 [growth arrest and DNA damage inducibility]). C/EBPs bind a consensus DNA sequence (A/G)TTGCG(C/T)AA(C/T) through their basic regions either as homodimers or as heterodimers with other family members via their leucine zipper motifs, as previously reviewed [14]. C/EBPζ lacks a functional DNA binding domain, and heterodimerization with other C/EBP proteins negatively regulates their DNA binding activity [15]. C/EBPs can also interact with c-Fos and CREB/ATF (cAMP response element binding/activating transcription factors) to form heterodimers that do not bind to consensus C/EBP sites [16]. Multiple isoforms of C/EBPα and C/EBPβ exist that are products of translational initiation at internal AUG codons. C/EBPα has two isoforms, p42 and p30. The *N*-terminally deleted p30 C/EBPα form does not function as a transcriptional activator. C/EBPβ, conversely, has four internal AUG codons that result in 38-, 35-, 21-, and 14-kDa proteins, respectively. C/EBPβ is also referred to as nuclear factor interleukin-6 in human and liver-enriched activating protein, in rodents. A 21-kDa liver inhibitory protein isoform also exists as a proteolytic cleavage product of full-length C/EBPβ [14], [16]. C/EBPα has also been shown to be regulated during the course of the cell cycle by binding to CDK2 and CDK4 (cyclin-dependent kinases) and inhibiting their activity [17], [18]. C/EBPs are also known to cooperate with nuclear factor for activated T-cells (NFAT) to control expression from promoters such as calcineurin regulatory protein RCAN1-4 [19], and they also act together as a composite enhancer complex [20], [21].

The NFAT family of transcription factors consists of five proteins that are related to the REL-NF-κB family. These include NFAT1 (also known as NFATc2 or NFATp), NFAT2 (NFATc1 or NFATc), NFAT3 (NFATc4), NFAT4 (NFATc3 or NFATx), and NFAT5 (tonicity enhancer binding protein, TonEBP), as previously reviewed [22]. They play a central role in transcriptional regulation of genes associated with immune responses in both lymphoid and myeloid lineages [23], [24]. NFATs show tissue-specific distribution and function. NFAT1 and NFAT2 are expressed in both T-cells and macrophage-lineage cells, where they play crucial regulatory roles in expression of genes critical for the immune response. The HIV-1 LTR has recognition sites for NFAT1 and NFAT2 that are crucial for HIV-1 replication in primary CD4^+^ T-cells [25], [26]. The isoform NFAT5 has been shown to regulate HIV-1 in primary monocytes by binding to a highly conserved LTR site that overlaps with the NF-κB sites [27]. Moreover, a binding site downstream of the TAR (transactivation responsive element) region has been shown to bind NFATs and control HIV-1 gene expression [28]. Highly phosphorylated inactive forms of NFAT have been shown to reside in the cytoplasm, and activation via receptors that are linked to calcium (Ca^2+^) mobilization and to heterotrimeric G protein–coupled receptors results in dephosphorylation and nuclear translocation of NFATs [29]. The activation of NFATs has been shown to be regulated by several factors, including calcineurin, the calcium/calmodulin-regulated serine/threonine phosphatase, and multiple inducible and constitutive kinases [24]. NFAT proteins have a highly conserved DNA-binding domain referred to as the Rel-homology domain. The regulatory domain contains multiple serine-rich regions that are phosphorylated by NFAT kinases, including casein kinase 1 (CK-1), glycogen synthase kinase 3 (GSK3), and dual-specificity tyrosine-phosphorylation-regulated kinase (DYRK), as previously reviewed [22], [23]. In addition to the kinases, NFATs can be negatively regulated by the noncoding repressor of NFAT (NRON) and caspase 3 and also by a number of scaffold proteins. NFATs by themselves are known to be relatively poor transcription activators but they are known to interact with and recruit other TFs that are potent activators, including AP-1 [30], and C/EBPα [19], [21], [31].

We identified a novel downstream cis-acting element in the HIV-1 LTR that is utilized by C/EBP and NFAT family members to positively and selectively control transcription in cells of the monocyte-macrophage lineage. These results provide additional evidence pointing to the importance of downstream LTR binding sites with respect to nucleosome positioning within the context of the viral promoter.

## Materials and Methods

### Cell Culture and Treatments

The human histiocytic lymphoma cell line U-937 (ATCC [American Tissue Culture Collection], CRL-1593.2) is a promonocytic cell line that was used as a model of cells in the monocytic lineage and will be referred to herein as U-937 cells [32], U1/HIV-1 cells [33] and Jurkat CD4^+^ T-cells (ATCC, TIB-152) were cultured and maintained in Roswell Park Memorial Institute (RPMI)-1640 (Cellgro; Mediatech, Manassas, VA) supplemented with penicillin (100 U/mL), streptomycin (100 µg/mL), sodium bicarbonate (0.15%) (Cellgro), and fetal bovine serum (10%) (GermCell; Gemini Bio-products, West Sacramento, CA). HEK 293T cells (ATCC, CRL-11268) were cultured in Dulbecco minimal essential medium (Cellgro) supplemented with penicillin (100 U/mL), streptomycin (100 µg/mL), sodium bicarbonate (0.15%) (Cellgro), and fetal bovine serum (10%) (GemCell). U-937 and U1/HIV-1 cells were stimulated for 12 hours, where indicated, with human interleukin-6 recombinant protein (20 ng/mL; ebioscience, San Diego, CA) prior to harvesting. U-937 and U1/HIV-1 cells were also treated with 1 µM Cyclosporine A (Cell Signaling Technology, Boston, MA) for 24 hours followed by overnight treatment with IL-6, where indicated.

### Nuclear Extract Preparation and Electrophoretic Mobility Shift (EMS) Analyses

Small-scale nuclear extracts were prepared from low-passage, exponentially growing cells. Briefly, cells (1×10^7^) were collected by centrifugation, washed once with ice-cold 1× Dulbecco phosphate-buffered saline (Mediatech), and lysed in ice-cold lysis buffer [HEPES (10 mM) pH 7.9, KCl (10 mM), EDTA (0.1 mM), EGTA (0.1 mM), octylphenoxypolyethoxyethanol (IGEPAL; Rhodia) (0.4%), dithiothreitol (DTT; 1 mM), phenylmethylsulfonyl fluoride (PMSF; 0.5 mM), and Halt protease inhibitor cocktail (1∶100; Thermo Scientific, Rockford, IL)]. After centrifugation (1000×*g*), the supernatant (cytoplasmic extract) was discarded. The pelleted nuclei were gently resuspended in nuclear extract buffer [HEPES (20 mM), NaCl (0.4 M), EDTA (1 mM), EGTA (1 mM), DTT (1 mM), PMSF (1 mM), and Halt protease inhibitor cocktail (1∶100)], shaken vigorously on a rocker for 30 min at 4°C, and subjected to centrifugation for 10 min (14,000×*g*). The supernatant (nuclear extract) was then dialyzed two times for 45 minutes using minidialysis units (Thermo Scientific, 2000 molecular weight cutoff) in dialysis buffer [HEPES (20 mM), KCl (100 mM), EDTA (0.2 mM), glycerol (20%), DTT (1 mM), and PMSF (1 mM)]. The nuclear extract was recovered from the dialysis units and transferred to an Eppendorf tube and stored at −80°C in small aliquots. The protein concentration was determined by Bradford assay as described by the manufacturer (Bio-Rad, Hercules, CA).

The sequences used to generate double-stranded, gel-purified oligonucleotides for EMS analysis as previously described [34] included C/EBP US2, AGCATTTCATCACAT; C/EBP US1, TGCAGCTTTCTACAAGGG; DS3, AGTCAGTGTGGAAAATCTCT; DS3-9C, AGTCAGTGTGCAAAATCTCT; DS3-11C, AGTCAGTGTGGACAATCTCT; DS3-12C, AGTCAGTGTGGAACATCTCT; DS3-5C, AGTCAGCGTGGAAAATCTCT; NFAT consensus, CGCCCAAAGAGGAAATGTTTCATA; and consensus subtype B Sp site III, AGGGAGGCGTGGCCTG. Oligonucleotides were synthesized by Integrated DNA Technologies (Coralville, IA) as previously described [34]. For antibody supershift/abrogation analysis, the indicated antibody (2 µg per reaction) (Santa Cruz Biotechnologies, Santa Cruz, CA) was added to the reaction and incubated for 30 min at room temperature prior to addition of the labeled probe.

### Chromatin Immunoprecipitation Assays

U1/HIV-1 cells were either stimulated with IL-6 or treated with a combination of cyclosporine A (1 µM) and IL-6 (20 ng/ml) as described earlier. At the time of harvest, the cells were cross-linked with 16% formaldehyde achieving a final concentration of 1%. The cells were incubated with gentle rocking at room temperature for 10 min, transferred to centrifuge tube on ice, collected by centrifugation at 1,100 rpm for 5 min, and washed twice with cold phosphate-buffered saline (PBS). Another wash was performed with PBS containing HALT protease inhibitor cocktail (Thermo Scientific, Pittsburgh PA). Subsequent steps of chromatin immunoprecipitation were performed as previously described [35]. Soluble chromatin (50 µg) was used for each immunoprecipitation along with 5–10 µg of relevant antibody. Four µl of immunoprecipitated DNA or input DNA was subjected to PCR in a final volume of 50 µl using GoTaq hot start polymerase as previously described by the manufacturer (Promega, Madison, WI). Each sample was subjected to 95°C for 5 min followed by 40 cycles at 95°C for 30 s 56°C for 30 s, and 72°C for 30 s. The final extension was performed at 72°C for 5 min. The following primers were used to amplify the +91 to +238 region (148 base pairs) of the HIV-1 LTR that encompasses the TFBS (DS3) exclusively: 5′ – GCTTCAAGTAGTGTGTGCCCGT- 3′ and 5′-TGCGTCGAGAGAGCTCCTCTG –3′. The DNA immunoprecipitated with RNA polymerase-II antibody was also amplified using primers for the GAPDH promoter (Thermo Scientific, Pittsburgh PA), as a positive control for the assay. Each PCR was run on 2% agarose gel containing ethidium bromide and analyzed using Alphaimager.

### Antibodies

The anti-C/EBPα (C-18, sc-9314×), anti-C/EBPβ (C-19, sc-150×), anti-NFATc2 (M-300, sc-13034×), anti-NFATc1 (7A6, sc-7294×), anti-NFATc1 (H-110, sc-13030×), anti-NFAT5 (sc-13035×), and control rabbit IgG (sc-2027) were obtained from Santa-cruz Biotechnology, Dallas, Texas. RNA pol II antibody (61082) was obtained from Active Motif, Carlsbad, CA. Mouse β-actin and anti-FLAG monoclonal antibodies were obtained from Sigma-Aldrich, St. Louis, MO.

### Cloning and Site-directed Mutagenesis

pGL3-based HIV-1 LAI-LTR luciferase reporter vector was used as a template to generate the DS3 knockout (9C) mutant (involving a G-to-C change at position number 9 in the DS3 binding site) utilizing the QuickChange II site-directed mutagenesis procedure (Stratagene, Cedar Creek, TX). The terminal FLAG-tagged C/EBPβ expression vector was constructed by cloning human C/EBPβ cDNA (Thermo Scientific Mammalian Gene Collection Clones, Waltham, MA) in pFLAG-CMV2 vector (Sigma-Aldrich, St. Louis, MO) between *Hin*dIII and *Bam*HI restriction endonuclease sites in the multiple cloning site. The vector was verified by sequencing and expression was confirmed by western immunoblotting with anti-FLAG antibody (Sigma-Aldrich).

### Transient Transfections

Exponentially growing U-937 cells or Jurkat T-cells were plated into 12-well tissue culture plates on the day of transfection at a concentration of 2×10^5^ cells/mL. Transient transfections were performed using Lipofectamine LTX with Plus Reagent as recommended by the manufacturer (Life Technologies, Carlsbad, CA). Luciferase expression constructs (1 µg) [HIV-1 LAI-LTR-luc or DS3 knockout (9C)-luc] and 50 ng of pRL-TK *Renilla* luciferase internal control vector were co-transfected in U-937 cells or Jurkat T-cells. For U-937 cells, interleukin-6 (20 ng/mL) stimulation was done 3 hours after transfection. The cells were harvested 24 hours after transfection and assayed using the dual-luciferase assay system as described by the manufacturer (Promega, Madison, WI). Firefly luminescence was normalized to *Renilla* luminescence. Each value shown represents the average of duplicate or triplicate transfection reactions and was representative of at least three independent experiments. Error bars indicate the standard error. HEK 293T cells were transfected using Xtreme Gene HP DNA transfection reagent as described by the manufacturer (Roche, Indianapolis, IN). Cells were transfected with either the vector (pFLAG-CMV2) or C/EBPβ expression vector (pFLAG-CMV2-C/EBPβ). Cells were harvested for nuclear extract preparation 24 hours after transfection and used for EMS analysis.

### Western Immunoblot Analysis

U-937 cells (1×10^6^ cells/well) were seeded in a six-well plate and were either left untreated or stimulated with interleukin-6 (20 ng/mL) overnight before harvesting. The cells were washed with ice-cold phosphate-buffered saline and lysates were made in radioimmunoprecipitation assay buffer (Thermo Scientific, Pittsburgh PA). Protein estimation was performed using the Bradford assay and samples were diluted 1∶2 with Laemmli Sample Buffer (Bio-Rad). Protein (30 µg per lane) was loaded and blotted using specified antibodies.

### Statistical Analysis

Data were generated from at least three replicate experiments. The statistical analysis was conducted by student t-test and a p value of <0.05 was considered to be statistically significant.

## Results

### DS3, a Binding Site Downstream of the Transcription Start Site, is Well Conserved Within HIV-1 Subtype B and Represented in Other Subtypes

**Figure 1:**
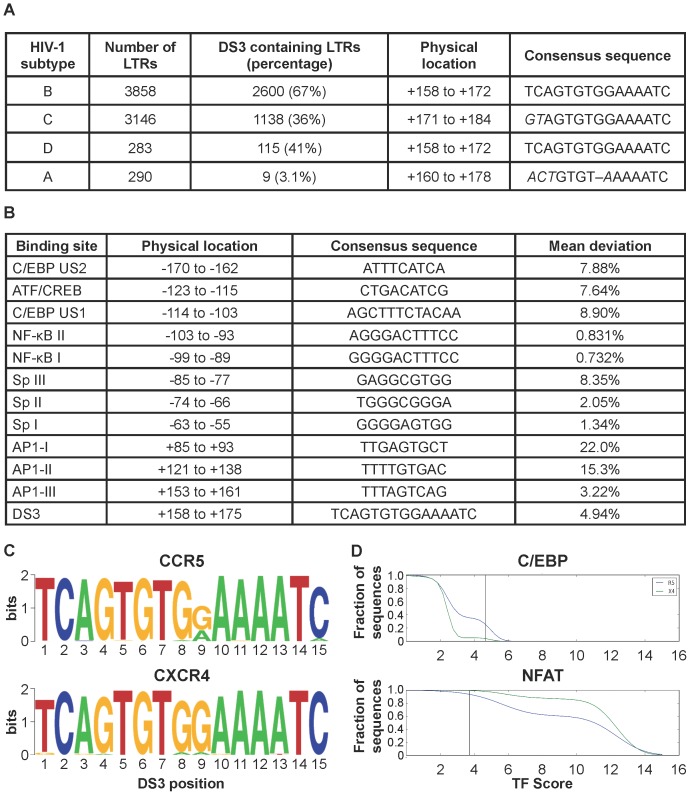
Physical position and consensus sequence comparison of DS3 among HIV-1 subtypes A, B, C, and D and differential binding phenotype in CXCR4 and CCR5-utilizing viruses. (**A**) Genbank and LANL were searched for HIV-1 LTR sequences from subtypes A, B, C, and D resulting in 7,577 sequences. The MUSCLE alignment tool and TRANSFAC software were used to identify a predicted binding site for C/EBP factors from+158 to+172 with respect to the start site. This region was then used to construct a new consensus binding sequence for each of the four predominant subtypes in the dataset. (**B**) Comparison of nucleotide sequence deviations, from the consensus subtype B configuration, in important transcription factor binding sites in the LTR both upstream and downstream of the start site. (**C**) Sequence logos generated from 1,832 sequences which contained both LTR and envelope V3 sequence grouped by CXCR4 and CCR5 coreceptor utiliziation using WebPSSM. The differences observed were not statistically significant (Fischer’s exact test). (**D**) Survival function (1 - cdf) of the distribution of Jaspar binding scores for the NFAT and CEBP sites of the DS3 region for sequences in C grouped by predicted coreceptor utilization. The vertical black bars represent the binding threshold with a false positive rate of 0.01.

Studies were initiated by compiling HIV-1 sequences from the Genbank repository. A local blast search was used against a database developed with the Los Alamos database Curated Alignment Set (http://www.hiv.lanl.gov/content/sequence/HIV/mainpage.html) to identify Genbank records with a full-length LTR sequence. As of 10/01/2013 there were a total of 510,217 HIV sequences. To ensure that each sequence was from a different patient, only a single LTR was selected from each referenced paper in the Genbank records, which resulted in a total of 9,712 full-length LTR sequences from all HIV subtypes. HIV-1 LTRs from subtypes A, B, C, and D totaling 7,577 sequences were then analyzed using the MUSCLE alignment tool to align these sequences. TRANSFAC software (http://www.biobase-international.com/product/transcription-factor-binding-sites) was then used to examine predicted TFBSs for known TFs. Utilizing this approach, a predicted binding site for C/EBP factors was identified from+158 to+172 with respect to the start site (+1) in 2600 (67%) of the 3858 subtype B sequences analyzed (Figure 1A). This region was then used to construct a new consensus binding sequence for each of the four predominant subtypes in our dataset (Figure 1A).

The next focal point that was addressed centered on the conservation level of this newly identified site (DS3) when compared with other well-characterized upstream and downstream TFBSs in subtype B LTRs. This was performed by measuring the average percentage of point mutations from ConB for the C/EBP US2, US1, and DS3; NF-κB sites I and II; Sp1 sites I, II, and III; the ATF/CREB site; and AP1-I, -II, and -III sites. For each sequence and each binding site, the best matching location was identified, within 10 nucleotides of the expected position, and the average deviation from ConB was calculated. Then that number was averaged for the entire binding site to give the mean percentage nonconsensus configuration of each site (Figure 1B). Based on this analysis, it was determined that DS3 was as well conserved as some of the crucial upstream binding sites, including upstream Sp binding sites and the downstream binding sites for AP TFs, which are important regulatory sites in HIV-1 transcription. Given C/EBP factors have been previously identified to have differential function in T- cells versus cells of the monocyte-macrophage lineage [11], the DS3 sequence was examined for differential genotypic signature in viruses that preferentially infect these two cell types. The Los Alamos National Laboratory (LANL) and Genbank databases were queried for subtype B sequences that have been characterized as T-cell tropic and macrophage tropic isolates; but the number of sequences was very low. Thus, an alternative approach was taken in which sequences that had both co-linear LTR and envelope in these two databases were identified. This totaled 1,832 sequences. The V3 portion of the envelope was then analyzed using WebPSSM [36], a bioinformatic tool for predicting HIV-1 coreceptor usage from the amino acid sequence of the third variable loop (V3). The co-linear viral LTR sequences were then identified as either CXCR4- or CCR5-utilizing viruses to loosely group them as T-cell (X4) versus macrophage (R5) tropic viruses. It is understood that many X4 viruses infect cell types other than T-cells and many R5 viruses have been shown to infect T-cells. Sequence logos were generated for DS3 in these two groups of sequences to search for differences [37]. It was found that the logos generated had some differences at position 9 with respect to the number of sequences that contained a G at this position but this difference was found to be statistically insignificant using Fisher’s exact test (p = 0.3, Figure 1C) with respect to amount of sequence variation between X4 and R5-utilizing viruses. To investigate the potential binding phenotype effect of a G or A at position 9, the Jaspar transcription factor tool [38] was used to determine the predicted binding strength of all 1832 sequences described above for NFAT and C/EBP (Figure 1D). Using a chi-squared test, it was determined that a larger fraction of the R5 sequences have a predicted CEBP binding site relative to X4 (p<0.001) due to the increase in the G variation observed at this position.

### DS3 Competes with C/EBP Binding Sites US2 and US1 *in vitro*

**Figure 2:**
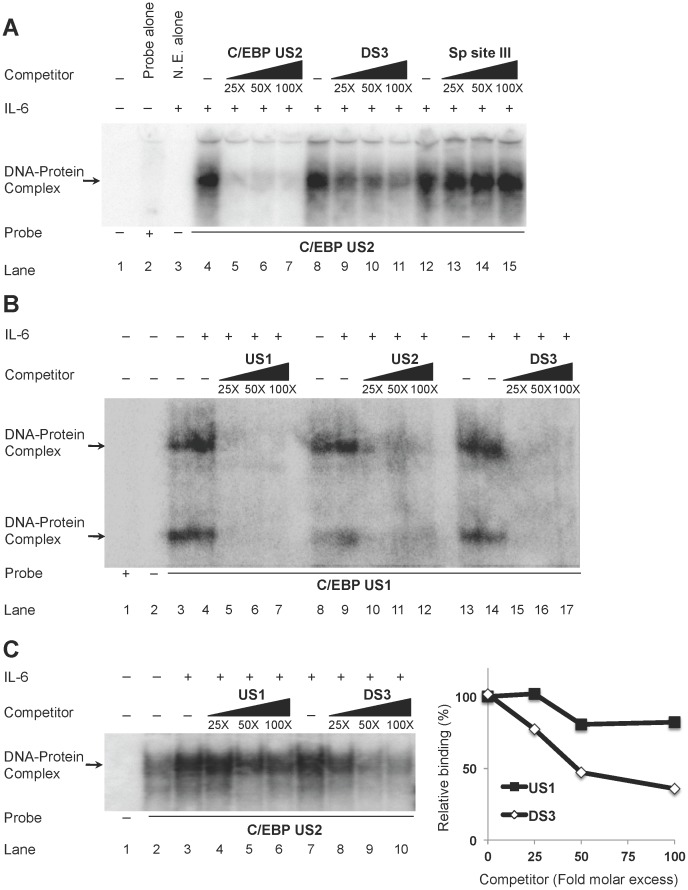
DS3 competes for US2 and US1 C/EBP complexes. Competitive EMS analyses were performed with ^32^P-labeled oligonucleotides (indicated below the figures) and incubated with nuclear extract from U-937 cells (unstimulated or stimulated with IL-6 (20 ng/mL), as indicated. (**A**) IL-6-stimulated U-937 nuclear extracts were incubated with labeled C/EBP US2 probe and cold competition was performed with molar excess of either unlabeled US2 (homologous competition) or unlabeled DS3 or Sp site III oligonucleotides (heterologous competition). (**B**) U-937 nuclear extracts stimulated with IL-6 were incubated with labeled C/EBP US1 oligonucleotide and competition was performed using unlabeled oligonucleotides representing C/EBP US1 (homologous competition, lanes 5–7); C/EBP US2 (heterologous competition, lanes 10–12); or DS3 (heterologous competition, lanes 15–17). (**C**) U-937 nuclear extracts stimulated with IL-6 were incubated with labeled C/EBP US2 oligonucleotide and competition was performed using unlabeled oligonucleotides representing C/EBP US1 (lanes 4–6) or DS3 (lanes 8–10) (right panel); densitometry quantitation is shown in the right panel.

To determine whether the predicted DS3 site was functional in terms of its ability to bind C/EBP factors, EMS analyses were performed using U-937 cell nuclear extracts. Because the C/EBP transcription factor family plays a critical role in transcriptional regulation in cells of the monocyte-macrophage lineage compared with many other types of cells targeted by HIV-1, U-937 cells were selected to model this interaction because of their monoblastoid cell heritage and because they have been extensively used in studies focused on HIV-1 gene regulation in monocytic cells. HIV-1 subtype B LTR upstream C/EBP binding site 2 (HIV-1 US2), which has been identified as a relatively high-affinity C/EBP binding site, was radiolabeled and used as a probe and incubated with U-937 nuclear extracts (Figure S1). Because treatment with interleukin-6 (IL-6) was known to activate C/EBP proteins [39], IL-6-stimulated U-937 nuclear extracts were used to show upregulation in nuclear levels of C/EBP protein (Figure S1A), which was also reflected in a more robust DNA-protein complex formation observed in EMS analyses (Figure S1B; compare lanes 2 and 5). The identities of the complexes observed were verified by homologous competition and using an antibody against C/EBPβ (Figure S1B; lanes 3 and 4 and lanes 6 and 7). When competitive EMS analysis was performed using increasing molar excess of unlabeled DS3, DS3 was able to compete for protein complex formed by the C/EBP binding site US2 (Figure 2A, compare lanes 9–11 with lanes 5–7); however, no competition was observed when unlabeled molar excess of an unrelated site (Sp site III) oligonucleotide was utilized (Figure 2A, compare lanes 13–15 with lanes 5–7). This analysis was performed because of the concern that the competition observed might have been due to excess unlabeled DNA in the binding reaction, helping to support the conclusion that the interactions at DS3 were specific. Because the competition observed with DS3 for C/EBP complexes with a high-affinity US2 site was low, a well-established C/EBP binding site of much lower binding affinity (US1) was evaluated to see how it compared with binding affinity of DS3 with respect to binding to C/EBP. To this end, IL-6-stimulated U-937 nuclear extracts were incubated with radiolabeled C/EBP US1 and cold competitive analyses were performed using unlabeled US2 and DS3 (Figure 2B). DS3 was able to compete as efficiently as US2 for US1 C/EBP complexes, further reinforcing the observation that C/EBP proteins bind DS3. To compare the relative affinities of the three C/EBP binding sites in the LTR (US2, US1, and DS3), competitive EMS analyses with radiolabeled US2 using interleukin-6-stimulated U-937 extracts were performed and demonstrated that DS3 competition for C/EBP US2 complexes was much stronger as compared with competition by US1 (Figure 2C, left panel). Densitometry (Figure 2C, right panel) clearly showed that DS3 exhibited a preference for C/EBP-US2 complex formation as compared with C/EBP-US1 complex formation. Thus, it was concluded that the putative binding site (DS3) in HIV-1 LTR binds C/EBP isoforms in a promonocytic leukemic cell line and shows intermediate affinity for C/EBP as compared with a higher preference of US2 and low preference for US1.

### NFAT Isoforms Show Preferential Binding and Demonstrate Strong Affinity for the DS3 Downstream Element

**Figure 3:**
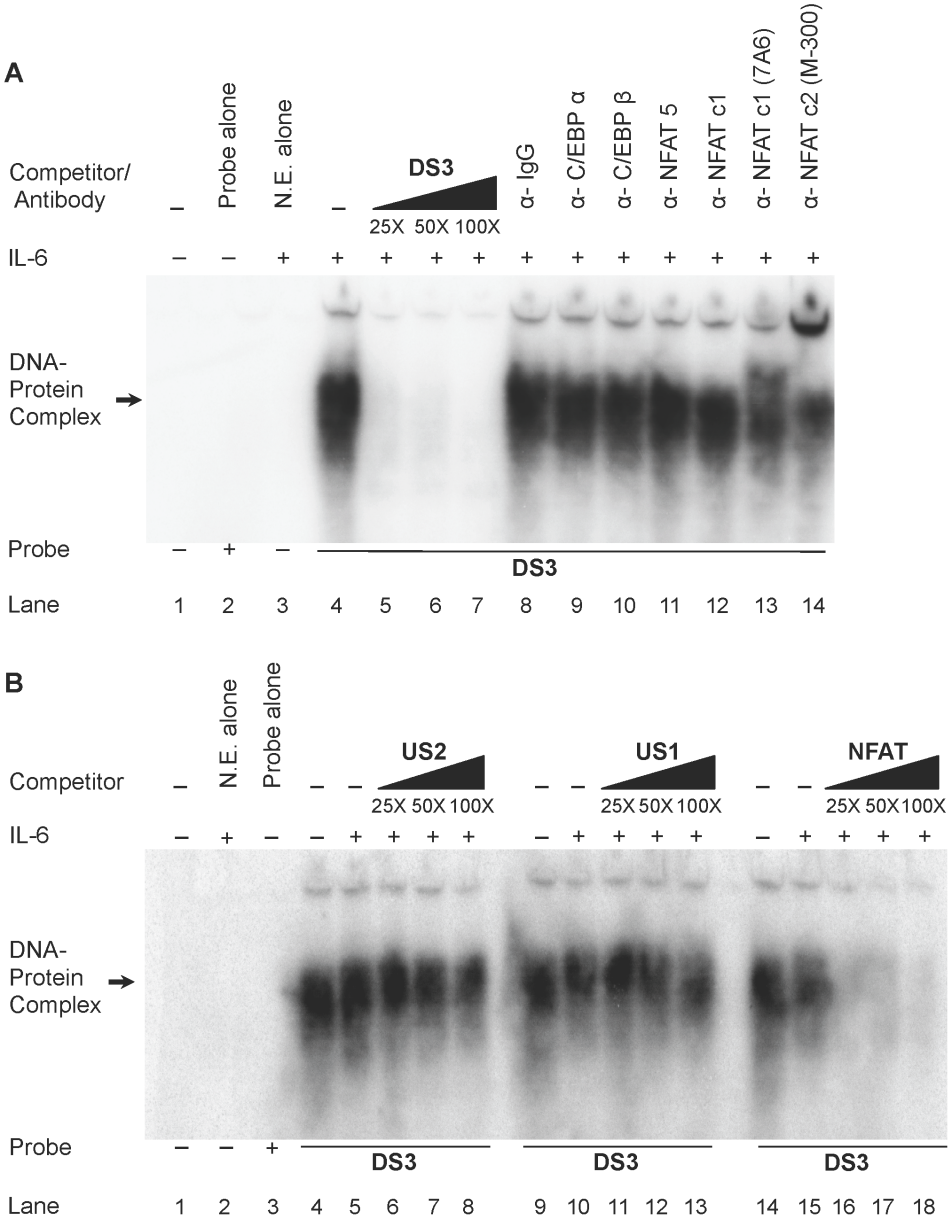
NFAT isoforms preferentially form complexes with DS3. (**A**) Nuclear extracts of IL-6-stimulated U-937 cells were incubated with labeled DS3 oligonucleotide and either homologous competition (lanes 5–7) or incubation with control IgG or specific antibody (lanes 8–14) was performed. (**B**) Nuclear extracts of IL-6-stimulated U-937 cells were incubated with labeled DS3 oligonucleotide and homologous competition was performed with unlabeled US2 (lanes 6–8), US1 (lanes 11–13), or consensus NFAT (lanes 16–18) oligonucleotides.

The region downstream of nucleosome 1 in the LTR is an important regulatory stretch and presents binding sites for a number of transcription factors including AP-1, NFAT, Sp1, and IRF (interferon-responsive factor) [9]. The TF interactions at these sites become particularly important in regulating transcription from the LTR in productively infected cells as nucleosome 1 is remodeled in the process and RNA polymerase subsequently moves ahead to generate HIV-1 transcripts. Previous reports have suggested the presence of NFAT in DNA-protein complexes that form with the downstream region [9], [28], and numerous reports have demonstrated that C/EBPβ and NFAT cooperate in driving gene expression from unrelated promoters such as the calcineurin regulatory protein RCAN1-4 [19] and secretory phospholipase A_2_ [21]. Other studies have also shown them to participate in a composite enhancer complex [20]. Taken together these findings suggest that these two well-characterized TFs may interface at DS3 to regulate HIV-1 transcription. Moreover, when one inspects the binding sites for NFAT in selected promoters such as interleukin-2, granulocyte-macrophage colony-stimulating factor enhancer, the NF-κB-like site, and the NFAT-GATA sites [30], NFAT can be shown to interact with the motif containing GGAAAXXXX; the residues GGAA are also present in the DS3 sequence CAGTGTGGAAAATC. These reports prompted the question as to whether NFAT isoforms also bind DS3, and if so, is the element a composite element with C/EBP or is there a competitive interaction at DS3 with C/EBP factors? To answer these questions, EMSAs were performed by incubating stimulated U-937 extracts with labeled DS3 oligonucleotide and relevant C/EBP and NFAT antibodies. Results show that NFATc2 antibody resulted in abrogation of the complex while other antibodies (C/EBPα, C/EBPβ, NFATc1 and NFAT5) did not (Figure 3A, compare lane 14 to lanes 9–13), indicating that NFAT isoforms, particularly the c2 isoform, preferentially bind DS3. Moreover, in competitive EMS studies, NFAT consensus oligonucleotide provided the best competition against DS3 complex formation as compared with US2 and US1 oligonucleotides (Figure 3B). This further reinforced the earlier observation that only NFAT c2 antibody abrogated DS3 complex formation. A reciprocal competitive EMS analysis was performed with consensus NFAT oligonucleotides reacted with DS3. Based on these experiments, DS3 was shown to be able to compete away the complex observed with NFAT oligonucleotide (Figure S2A), whereas C/EBP US2 and C/EBP US1 showed no competition with NFAT complexes (Figure S2B). Similarly, NFAT was unable to compete with C/EBP US2 complexes (Figure S2C). These results clearly demonstrate that the NFAT- and C/EBP-containing DNA-protein complexes are discrete and not the same.

Overall, these results lead to conclusion that NFATc2 isoforms preferentially occupy the DS3 binding site in the HIV-1 LTR and also that affinity of DS3 was optimized for NFATc2 isoforms. It was also concluded that the interactions at DS3 between NFATc2 and C/EBPs are competitive, as antibody supershift/abrogation was not observed with either of the respective antibodies.

### C/EBPα and C/EBPβ Bind DS3

**Figure 4:**
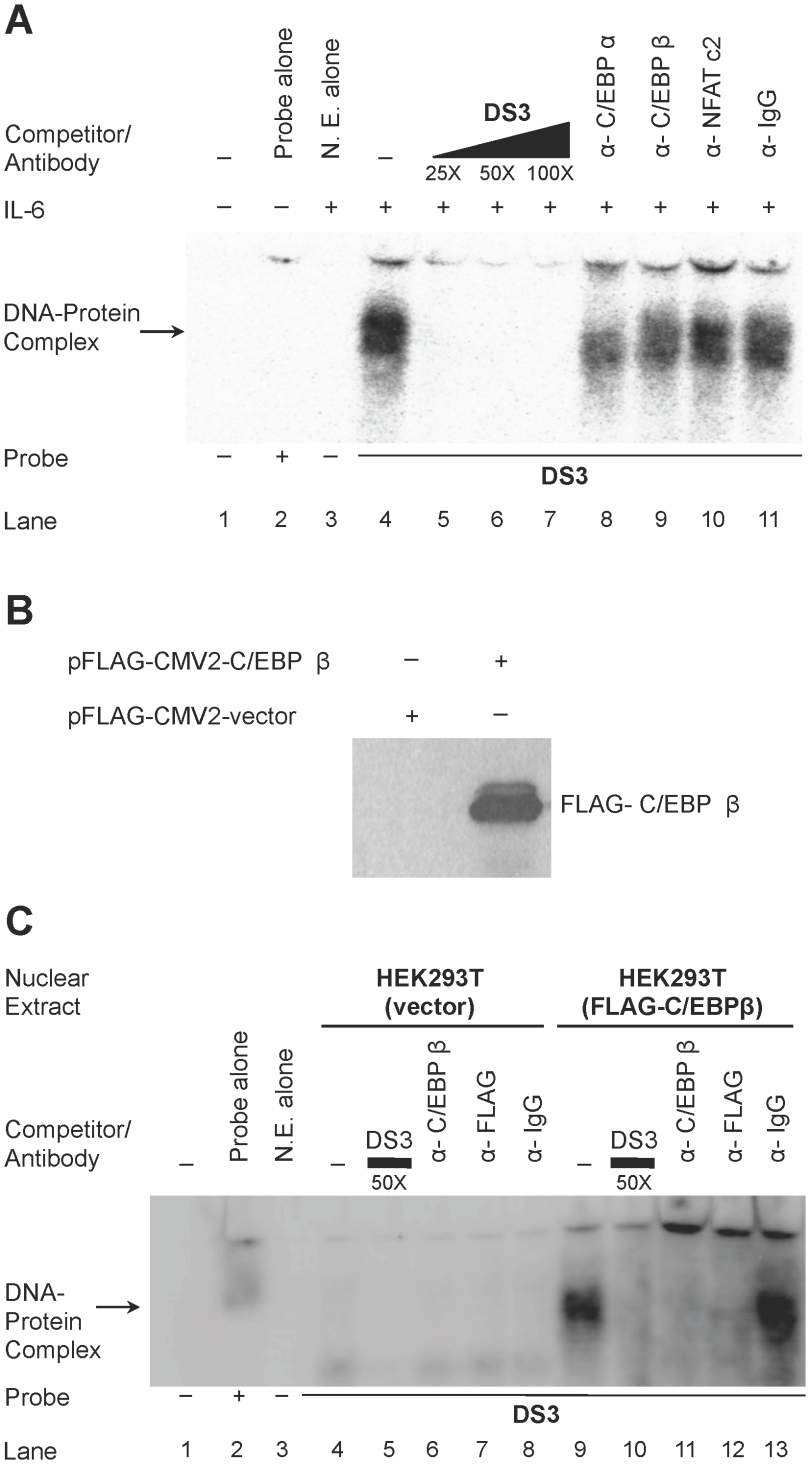
C/EBPα and C/EBPβ bind DS3 in the absence of NFAT. (**A**) Nuclear extracts of U-937 cells were incubated with labeled DS3 oligonucleotide (lane 4) and homologous cold competition was performed with unlabeled DS3 (lanes 5–7) or incubated with specified antibodies (lanes 8–11). (**B**) Western immunoblot showing overexpression of FLAG-tagged C/EBPβ in HEK 293T cells. (**C**) Nuclear extracts of HEK 293T transfected with either vector alone (lanes 4–8) or FLAG-C/EBPβ (lanes 9–13) were incubated with labeled DS3 and either homologous competition (lanes 6 and 10) or incubation was performed with indicated antibodies.

**Figure 5:**
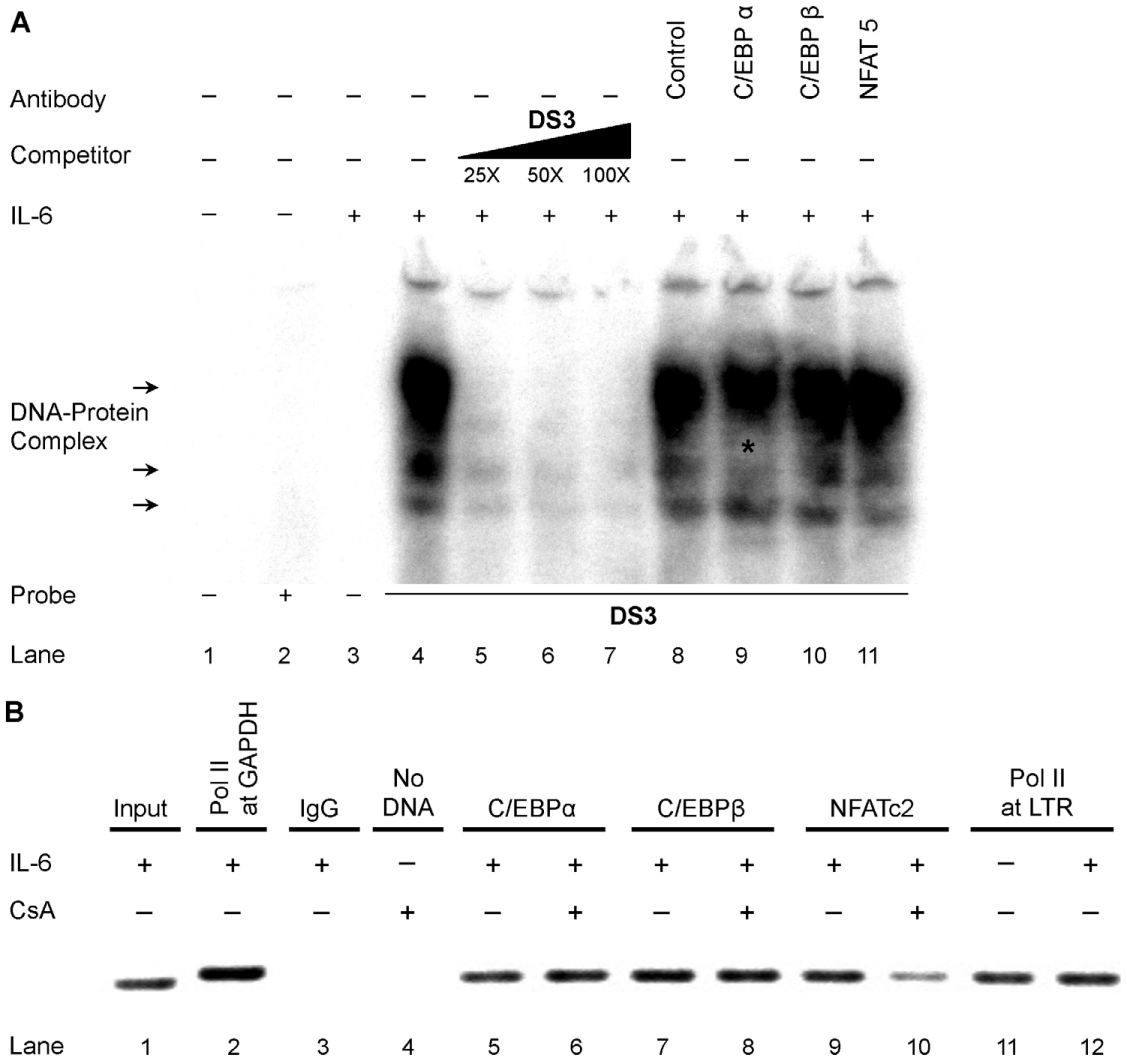
C/EBPα and C/EBPβ bind DS3. (**A**) Nuclear extracts of IL-6-stimulated U-937 cells were incubated with labeled DS3 oligonucleotide and homologous competition was performed with unlabeled DS3 (lanes 5–7) or incubated with specified antibodies (lanes 8–11). *Abrogation of the middle complex with C/EBPα antibody (lane 9). (**B**) ChIP was performed using 25 µg of soluble chromatin from U1/HIV-1 cells plus 5 µg of specific antibodies or non-specific isotype control antibody (IgG). DNA was detected using primers that amplify+91 to+238 downstream proviral LTR sequence. RNA Pol occupancy was also examined at the GAPDH promoter as a control for the assay in addition to the occupancy at the LTR. Input lane contains DNA that has not been immunoprecipitated.

NFATc1 through c4 are activated via stimulation of receptors linked to calcium signaling and G protein–coupled receptors (discussed in the introduction). Highly phosphorylated forms of NFATs have been shown to be inactive and stay in the cytoplasm and are only translocated to the nucleus as a result of dephosphorylation mediated by calcineurin phosphatase and other inducible or constitutive kinases. Because the results have suggested that the DS3 binding site was utilized competitively by C/EBP and NFAT isoforms and appeared to have higher relative affinity for NFAT isoforms, it was important to determine whether C/EBP preferentially occupies DS3 in situations where NFAT would be inactive and therefore remain in the cytoplasm. Cyclosporine A–treated U-937 nuclear extracts were used in EMS analyses to assess binding of C/EBP family members to DS3 (Figure 4A). As expected, cyclosporine A treatment resulted in reduced nuclear NFAT that was reflected in loss of NFAT binding to DS3 (Figure 4A, lane 10), and this resulted in binding of C/EBPα to DS3 as shown by abrogation of DS3 complexes in the EMS analysis (Figure 4A, lane 8). However, no abrogation was observed with C/EBPβ antibody (Figure 4A, lane 9). Studies were then performed to assess binding of C/EBPβ to DS3 using antibody supershift/abrogation analysis because DS3 was previously observed to compete for C/EBPβ complexes (Figure 2). This may be due to the lower affinity of C/EBPβ compared with the α isoform for the DS3 element. In this regard, overexpressed FLAG-tagged C/EBPβ produced in HEK 293T cells (with FLAG-C/EBPβ overexpression confirmed by western immunoblot, Figure 4B) and antibody supershift/abrogation analyses were performed with both anti-FLAG antibody and anti-C/EBPβ antibody. As shown (Figure 4C), both of these antibodies, but not the control IgG antibody, were able to abrogate the DS3 complexes, indicating that overexpressed C/EBPβ bound DS3 in these experimental conditions. Nuclear extracts from IL-6-stimulated U-937 cells were used in a higher percentage gel (6.5%) in order to resolve smaller complexes that were not being resolved previously and were potentially masked by larger NFAT complexes (Figure 5A). With these experimental conditions, a complex of lower abundance was observed being abrogated by C/EBPα antibody (Figure 5A, lane 9, highlighted by an asterisk). However, as expected there was no abrogation/shift with the NFAT5 antibody (lane 11). In addition, no change was observed with C/EBPβ antibody (lane 10), again suggesting that the α isoform exhibited a higher relative affinity for DS3 compared with the β isoform in U-937 cells. The binding phenotype of DS3 was also examined with nuclear extracts of T-cell origin that would have an abundant level of NFAT. Indeed, it was observed that NFAT antibody showed a robust abrogation of complex formation with DS3 in Jurkat T-cells (Figure S3). The abrogation observed was greater than that obtained with the NFATc2 antibody abrogation that was observed with DS3 complexes formed in U-937 cells (compare lane 14 in Figure 3A, with lane 4 in Figure S3).

The U1/HIV-1 cells, a cell line derived from U-937 cells chronically infected with HIV-1, is a standard model used to study the recruitment of transcription factors to the LTR and also to understand the transcriptional regulation of an integrated HIV-1 provirus [33]. Chromatin Immunoprecipitation (ChIP) was employed to analyze the binding of transcription factors to the LTR specifically to the DS3 region. ChIPs were performed with antibodies against C/EBPα, C/EBPβ, NFATc2, and RNA Pol II in IL-6-stimulated and IL-6- and CsA-treated conditions. C/EBPα, C/EBPβ, and NFATc2 factors bound the DS3 region in the integrated HIV-1 LTR (Figure 5B, lanes 5, 7 and 9). Moreover, additional treatment of these cells with CsA resulted in reduced NFATc2 binding (compare lanes 9 and 10), as expected and also a modest increase in C/EBPα binding (compare lanes 5 and 6). These results reinforced the observations seen in the in vitro setting using EMSA that in the integrated scenario, DS3 is an important site in terms of recruiting C/EBP and NFAT depending on the overall abundance of the proteins due to differences in stimulation.

### TF Binding at DS3 Positively Regulates Transcription from HIV-1 LTR

**Figure 6:**
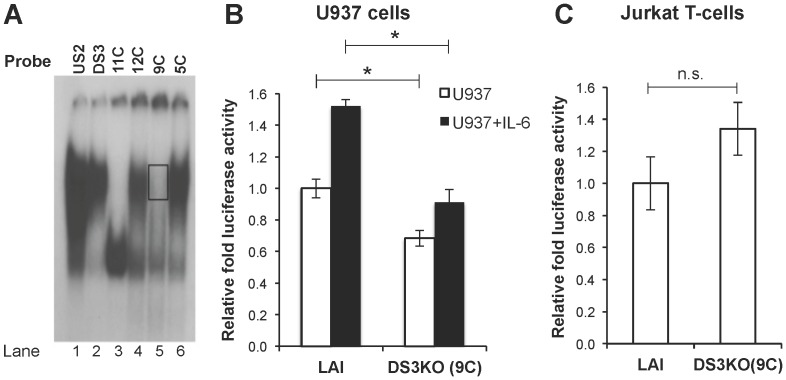
Functional impact of binding at DS3 in promonocytic U-937 and Jurkat T-cells. (**A**) EMS analyses were performed with labeled oligonucleotides corresponding to artificial mutations within DS3 and incubated with nuclear extracts from U-937 cells. (**B**) U-937 cells (1×10^6^) were co-transfected in triplicate with a luciferase reporter vector (1 µg) containing either the LAI HIV-1 LTR or DS3 knockout configuration and *Renilla* luciferase vector (50 ng). Relative luciferase activity was quantitated 24 hours after transfection and normalized to basal parental LAI levels (*p<0.05). (**C**) Jurkat T- cells (1×10^6^) were co-transfected in triplicate with a luciferase reporter vector (1 µg) containing either the LAI HIV-1 LTR or DS3 knockout configuration and *Renilla* luciferase vector (50 ng). Relative luciferase activity was quantitated 24 hours after transfection and normalized to basal parental LAI levels (n.s.- non-significant).

The DS3 element exhibited a higher relative affinity for NFATc2 than that observed with C/EBPα and β, and the binding appeared to be in a mutually exclusive manner. Such interactions have been previously described within the context of the HIV-1 promoter; for example, one AP-2 site has been shown to be embedded within two κB elements and AP-2 and NF-κB were shown to bind to this region in a mutually exclusive manner in Jurkat T-cells [40]. In this regard, the functional properties of this region of the viral promoter were examined with respect to LTR-directed transcription. TF binding at the U5 region of the LTR has been shown to be involved in regulating LTR-mediated transcription. For example, binding sites for AP-1, AP-3-like, DBF1, and Sp1 that are downstream of the transcriptional start site have been shown to regulate the basal transcriptional activity of the LTR because site-directed mutagenesis of these binding sites inactivated LTR-driven transcription [7]. Moreover, members of the NFAT family have previously been shown to control transcription via a binding site downstream of the TAR region [28]. Overall, these results established the importance of downstream binding sites in basal and activated LTR-directed transcription where their function may be governed by the chromatin structure of the integrated HIV-1 LTR. To this end, single-nucleotide mutations have been introduced into the DS3 binding site and the resultant LTR mutants have been examined for the ability to form DNA-protein complexes as compared with the C/EBP-US2 binding site. This process was pursued in order to select for a DS3 site variant that would display a dramatic reduction in DNA-protein complex formation as compared with the parental DS3 site while at the same time not showing any formation of new DNA-protein complexes not observed with the parental element. These results demonstrated that the nucleotide change at position 9 (G-to-C) resulted in abolishment of the complex and did not result in any new complex formation (Figure 6A, compare lane 5 with lanes 1 and 2). Based on this result, the 9C variant was selected as a DS3 binding site knockout configuration and this mutation was introduced into the expression vector containing the luciferase gene under the control of HIV-1 LTR (LAI strain). The construct was utilized in luciferase assays to determine the impact that DS3 has on LTR-directed transcription under basal as well as interleukin-6-stimulated conditions in U-937 cells (Figure 6B) as well as in Jurkat T-cells (Figure 6C). DS3 knockout (9C)-containing LTR was compromised in transcriptional activity from the LTR as compared with parental HIV-1 LAI LTR configuration in both basal and interleukin-6-stimulated conditions in U-937 cells (Figure 6B; relative reductions of 32% and 40.2% were observed as compared with LAI in basal and interleukin-6-activated conditions, respectively). Moreover, the LTR 9C variant DS3 knockout did not reach the transcriptional activity exhibited by basal LAI-LTR even in interleukin-6-activated conditions (Figure 6B). The functional impact of DS3 was examined in a similar way using Jurkat T-cells to compare the function of DS3 in a T-cell line (Figure 6C). As can be seen, a significant difference between the LTR-directed transcription in LAI and DS3KO configuration was not observed implying that the function of DS3 is selectively observed in a monocytic lineage and not in a T-cell lineage. These results highlight the differences in LTR-directed transcription in T-cells versus monocyte-macrophage lineage cells as has also been previously established [11]. Thus, these results suggest that DS3 plays a positive role in transcriptional activity of the HIV-1 LTR in U-937 cells selectively.

## Discussion

**Figure 7:**
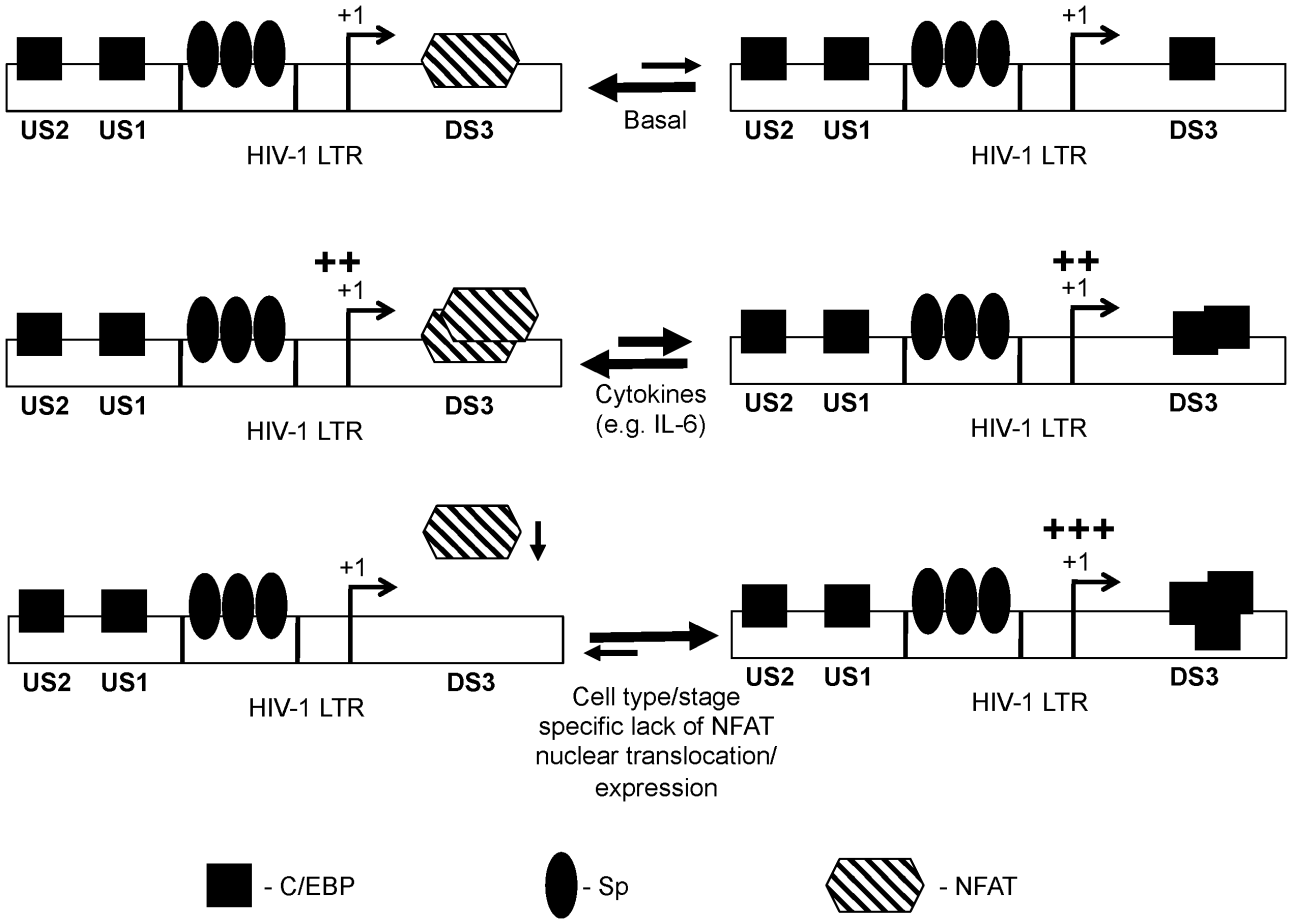
Experimental model. In basal conditions, DS3 stays in equilibrium between NFAT and C/EBP bound states. The affinity of NFATc2 is relatively higher than C/EBP isoforms for binding at DS3 (top panel; figures 3 and 4). However, stimulation with cytokines including IL-6 results in activation of C/EBP isoforms and, which is evident from their ability to bind the DS3 region (middle panel; figure 5). In conditions, where nuclear NFATc2 concentration was low, increased binding of C/EBPα to DS3 was possible (lower panel; figure 4 and 5).

In the HIV promoter a cluster of functional transcription factor binding sites downstream of the transcriptional start site has previously been defined, with binding sites including AP-1, NFAT, DBF/IRF, and Sp1 [9]. It is also known that upon transcriptional activation of the integrated LTR, a large region (∼255 nucleotides) downstream of the start site is free of nucleosomes due to concomitant chromatin remodeling of nucleosome 1 [5], [9]. In the present study, we characterized a downstream element in the LTR (+158 to +172, subtype B LTR as reference) that is bound by NFAT and C/EBP family members. More specifically, using gel shift analyses, it was shown that NFATc2 demonstrates a high relative affinity for the element while C/EBPα and C/EBPβ show relatively lower affinities (Figures 3, 4, 5). Moreover, it was establish that DS3 plays a positive role in LTR-directed transcriptional activity in U-937 cells and not in Jurkat T-cells (Figure 6). With these results, we propose an experimental model (Figure 7) that involves the preferential occupation of DS3 by NFAT under basal conditions based on its higher relative affinity for DS3 compared with C/EBP family members. However, both expression and activation of members of the NFAT and C/EBP families have been shown to be cell type- and signal-specific processes, and the relative occupancy of DS3 would exist as an equilibrium between NFAT and C/EBP bound states (Figure 7, top panel) with the equilibrium subject to alteration by these processes. The signals that induce activation of calcium signaling and nuclear translocation of NFATs would result in DS3 being preferentially occupied by NFATc2. However, specific signals including signals like IL-6, which is known to activate C/EBPs via the ras-ERK-MAPK (extracellular signal-related/mitogen-activated protein kinase) cascade [39], would result in a scenario where DS3 binding equilibrium is tilted towards increased C/EBP binding (Figure 7, middle and lower panels). Under IL-6-stimulated conditions too, NFATc2 is bound at DS3, this observation can be explained by some of the published studies that have shown that active C/EBP family members upregulate NFATc2 expression via binding to predicted sites in the NFATc2 promoter [21], [41]. Furthermore, conditions where nuclear NFAT has been shown to be low owing to its constant rephosphorylation and inactivation by cellular kinases such as CK1, GSK3, and DYRK, as well as by negative regulators of NFAT including NRON and caspase 3, one would expect C/EBP family members to bind DS3 and regulate transcriptional activity (Figure 7, bottom panel). We have tried to model these conditions by cyclosporine A treatment of the cells and did indeed observe an increase in C/EBPα binding to DS3 (Figure 4A and 5B). An abrogation of DS3 complex formation was observed with the C/EBPα antibody but not with the C/EBPβ antibody, implying that the α isoform demonstrated a stronger affinity than the β isoform in a scenario in which NFAT translocation was compromised.

The interactions at TFBS (DS3) were also analyzed using U1/HIV-1 cells that are chronically infected with HIV-1 and are a good model system to examine TF binding at selected regions of the LTR. ChIP experiments did recapitulate the data obtained with gel shift analyses and C/EBPα, C/EBPβ and NFATc2 occupancy was shown in this downstream region (Figure 5B). Measuring the transcriptional activity of the HIV-1 promoter after transient transfection of the LTR-driven luciferase constructs showed a positive correlation with binding events at the downstream element, with binding mutant (DS3KO) showing reduction in transcriptional activity from the LTR (Figure 6). Moreover, this effect was selective to the U-937 cells, as Jurkat T cells did not show any change in LTR-directed transcriptional activity with the DS3KO mutation (Figure 6C). These results indicated that DS3 is a positive regulatory element with regard to HIV-1 LTR transcriptional activity under both basal and activated conditions. This level of LTR-directed transcription may be representative of the earlier stages of viral infection prior to the expression of elevated levels of Tat or other viral factors involved in downstream events in viral replication.

The studies reported herein also extend the functional knowledge of NFATc2 in HIV-1 regulation in cells of the monocyte-macrophage lineage and also identify an additional element bound by members of the NFAT family. This binding site adds to the LTR sites that are regulated by NFATs [27]; another site resides within the NF-κB sites in the enhancer region of the LTR and has been shown to be utilized by the NFAT5 isoform. This is important as several studies [42], [43] have established the functions of the cyclosporine A–sensitive isoform of NFAT (NFATc2, otherwise known as NFAT1) predominantly in the T-cell lineage, including regulatory T cells, helper T_H_17 cells, CD8^+^ T cells, and also in dendritic cells and mast cells [22]. Other recent studies have also shown that NFATc2 was expressed in monocyte lineage cells including the murine monocytic cell line RAW264.7 and the human acute monocytic leukemia cell line THP-1 [44], and also in bone marrow–derived macrophages [45].

Upon integration into the cellular genome, the HIV-1 proviral DNA packaged into chromatin and nucleosomes is positioned precisely such that two nucleosome-free regions are established (−254 to +2 and +156 to +266) [9], [46], [47]. The first region has been shown to be associated with the promoter-enhancer elements in the U3 region of the viral LTR. The second region has been associated with the end of the U5 region and primer binding site. The binding site DS3 is located in the second nucleosome-free region, close to the 3′ boundary of nucleosome-1 (nuc-1), which prompted testing the functional role of this element in LTR-directed transcription. The region downstream of the start site in the HIV-1 LTR has been shown previously to participate in TF binding events that control LTR activity [8], [48]. U-937 cells were selected to dissect the function of DS3 as these cells have been more sensitive to respond to activation signals like IL-6 that culminate in activation of TFs like C/EBP [3].

C/EBPs and NFATs are both known to participate in the process of chromatin remodeling and the ensuing transcriptional activation. Several reports, in fact, have suggested that chromatin remodeling might as well be the primary function of NFAT elements as even high-affinity NFAT-binding sites are comparatively poor transcriptional activators in the absence of the collaborating TFs that normally associate/cooperate with NFATs [24], [49], [50]. Examples of TFs that cooperate with NFATs include AP-1 and C/EBP, which act by recruiting both the histone acetyltransferases and ATP-dependent remodelers such as the SWI/SNF (switch/sucrose nonfermenting) family [49]–[54]. Most of these functions for NFAT have been defined in T cells. Only recently have investigators begun to appreciate their role in other cell types, including cells of the monocyte-macrophage lineage [22]. Similarly, C/EBP family members have been shown to interact with the chromatin remodeling machinery, and it has been well established that C/EBPs, including C/EBPβ, associate with CBP/p300, PCAF, and SWI/SNF remodeling factors [55]–[57]. Moreover, it is also known that C/EBPβ regulates chromatin architecture by interacting with coactivators [57]. For example, CBP and PCAF have been shown to be recruited to the HIV-1 LTR by C/EBPβ (liver-enriched activating protein), leading to synergistic LTR activation. However, C/EBPβ lacking the *N*-terminal region prevents chromatin remodeling [56]. Thus, in the context of HIV-1 replication in a chromatin environment, the DS3 element may play a positive regulatory role by recruiting either NFAT or C/EBP depending on the relative availability and/or activation status of the particular TF. For instance, in an NFAT-bound state, the element would be projected to recruit selected TFs such as AP-1 that would result in recruitment of the chromatin remodeling enzymes and the eventual activation of transcription. In the case of the C/EBP-bound state, C/EBPs could recruit similar remodeling machinery to the LTR, either directly by interacting [58]–[61] with the components of chromatin machinery or indirectly by interacting with crucial viral proteins such as Tat [62]–[64], which would successfully bring the transcriptional activators to the downstream element.

With regard to the ability of C/EBP factors to regulate the LTR from downstream binding site cues can also be taken from simian immunodeficiency virus studies in which we have previously described downstream elements with which C/EBPs are able to interact and that control the rate of transcription [65], [66]. As evident from our functional studies, DS3 by itself does not drastically affect the extent of transcription but likely regulates recruitment at the initial stages of RNA polymerase progression that regulate its processivity. These events may be especially relevant in the case of HIV-1 because low-level transcriptional events, in the absence of key transactivators such as Tat, are sufficient to guide the early stages of viral replication; low-level viral transcription from the integrated provirus in privileged sites such as the brain and the bone marrow, within cells of the monocyte-macrophage lineage, would then wait for the activation cues that would lead to activation of persistent or latent proviral DNA. Future studies will assess the impact of cellular differentiation states on how the utilization of the DS3 element may prove crucial for proviral DNA latency and activation.

## Supporting Information

Figure S1 **IL-6 stimulation of U-937 cells increases levels of C/EBPβ. (A)** Western immunoblot of U-937 cell nuclear extracts using C/EBPβ polyclonal antibody. **(B)** Competitive and supershift/abrogation EMS analyses were performed with ^32^P-labeled oligonucleotides (indicated below the figures) and incubated with nuclear extract from unstimulated or stimulated (IL-6; 20 ng/mL) U-937 cells as indicated. IL-6 stimulation resulted in increased binding to C/EBP US2 probe (compare lanes 2 and 5), and this enhanced complex formation was abrogated by addition of C/EBPβ antibody (lane 7).
(TIF)

Figure S2 **Reciprocal competitive EMS analysis to show DS3 complex specificity. (A)** Competitive EMS analysis was performed in which nuclear extracts from U-937 cells were incubated with labeled NFAT oligonucleotide and cold competition was performed with molar excess of DS3 oligonucleotide. **(B)** Competitive EMS analysis was performed with nuclear extracts from U-937 cells that were incubated with labeled NFAT oligonucleotide, and cold competition was performed with molar excess of unlabeled C/EBP US2 or C/EBP US1 oligonucleotides. **(C)** Competitive EMS analysis was performed with nuclear extracts from U-937 cells that were incubated with labeled C/EBP US2 oligonucleotide, and cold competition was performed with molar excess of unlabeled NFAT oligonucleotide.
(TIF)

Figure S3 **DS3 binds to NFAT isoforms in Jurkat T cells.** Nuclear extracts from Jurkat T cells were incubated with labeled DS3 oligonucleotide and supershift/abrogation analysis was performed with indicated antibodies.
(TIF)
