# Supplementary figures and images for: CCAAT Enhancer Binding Protein and Nuclear Factor of Activated T Cells Regulate HIV-1 LTR via a Novel Conserved Downstream Site in Cells of the Monocyte-Macrophage Lineage
Source: PLoS One. 2014 Feb 14;9(2):e88116. doi: 10.1371/journal.pone.0088116 (PMC3925103; doi:10.1371/journal.pone.0088116)

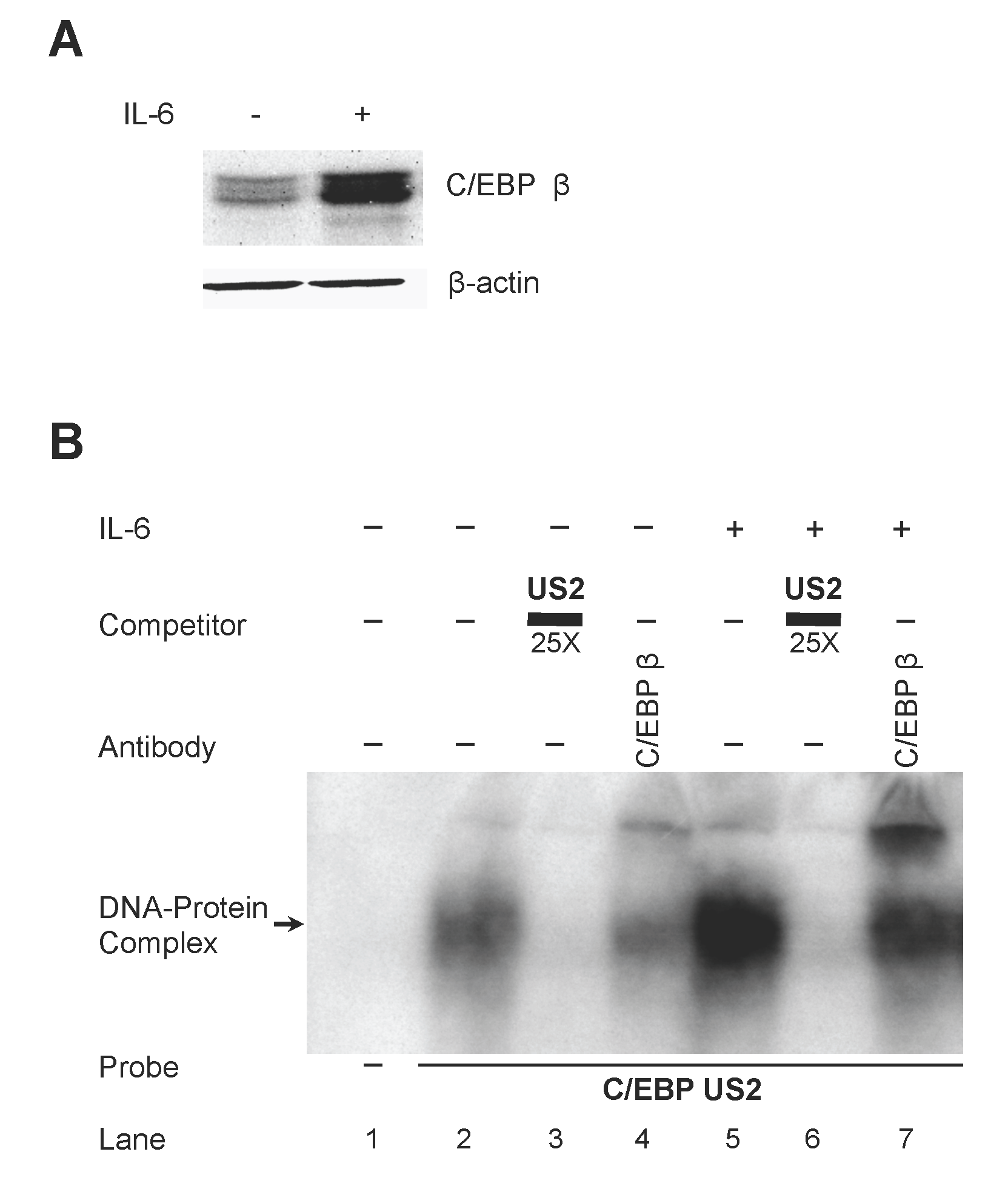

Supplement: Figure S1 — IL-6 stimulation of U-937 cells increases levels of C/EBPβ. (A) Western immunoblot of U-937 cell nuclear extracts using C/EBPβ polyclonal antibody. (B) Competitive and supershift/abrogation EMS analyses were performed with 32P-labeled oligonucleotides (indicated below the figures) and incubated with nuclear extract from unstimulated or stimulated (IL-6; 20 ng/mL) U-937 cells as indicated. IL-6 stimulation resulted in increased binding to C/EBP US2 probe (compare lanes 2 and 5), and this enhanced complex formation was abrogated by addition of C/EBPβ antibody (lane 7). (TIF) [file pone.0088116.s001.tif]

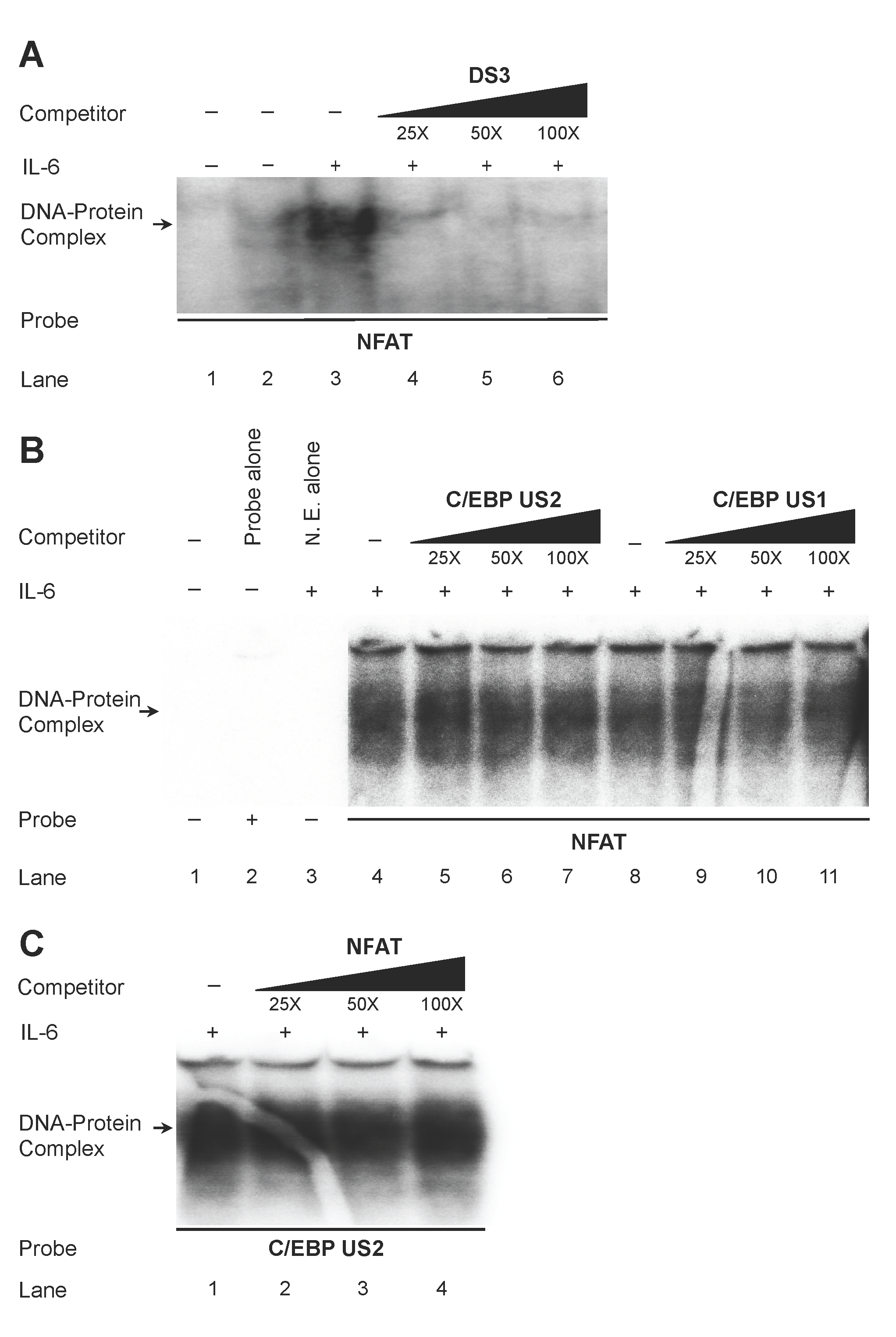

Supplement: Figure S2 — Reciprocal competitive EMS analysis to show DS3 complex specificity. (A) Competitive EMS analysis was performed in which nuclear extracts from U-937 cells were incubated with labeled NFAT oligonucleotide and cold competition was performed with molar excess of DS3 oligonucleotide. (B) Competitive EMS analysis was performed with nuclear extracts from U-937 cells that were incubated with labeled NFAT oligonucleotide, and cold competition was performed with molar excess of unlabeled C/EBP US2 or C/EBP US1 oligonucleotides. (C) Competitive EMS analysis was performed with nuclear extracts from U-937 cells that were incubated with labeled C/EBP US2 oligonucleotide, and cold competition was performed with molar excess of unlabeled NFAT oligonucleotide. (TIF) [file pone.0088116.s002.tif]

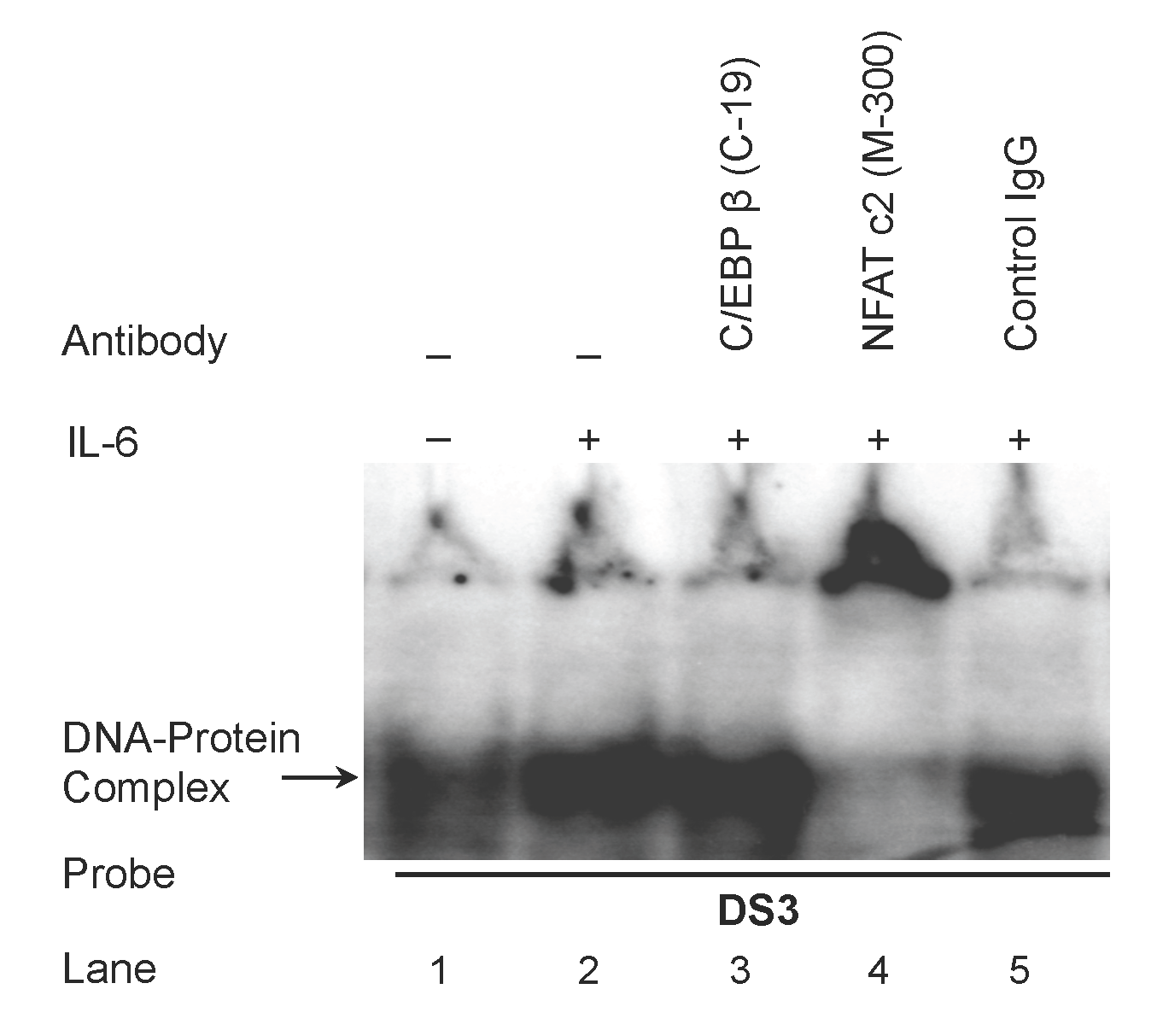

Supplement: Figure S3 — DS3 binds to NFAT isoforms in Jurkat T cells. Nuclear extracts from Jurkat T cells were incubated with labeled DS3 oligonucleotide and supershift/abrogation analysis was performed with indicated antibodies. (TIF) [file pone.0088116.s003.tif]
